# Melting temperature suppression of layered hybrid lead halide perovskites *via* organic ammonium cation branching†
†Electronic supplementary information (ESI) available. CCDC 1863836–1863839. For ESI and crystallographic data in CIF or other electronic format see DOI: 10.1039/c8sc03863e


**DOI:** 10.1039/c8sc03863e

**Published:** 2018-11-09

**Authors:** Tianyang Li, Wiley A. Dunlap-Shohl, Eric W. Reinheimer, Pierre Le Magueres, David B. Mitzi

**Affiliations:** a Department of Mechanical Engineering and Materials Science , Duke University , Durham , North Carolina 27708 , USA . Email: david.mitzi@duke.edu; b Department of Chemistry , Duke University , Durham , North Carolina 27708 , USA; c Rigaku Americas Corporation , 9009 New Tails Drive , The Woodland , Texas 77381 , USA

## Abstract

Hybrid organic–inorganic lead halide perovskites have attracted broad interest because of their unique optical and electronic properties, as well as good processability. Thermal properties of these materials, often overlooked, can provide additional critical information for developing new methods of thin film preparation using, for example, melt processing—*i.e.*, making films of hybrid perovskites by solidification of a thin layer of the melt liquid. We demonstrate that it is possible to tune the melting temperature of layered hybrid lead iodide perovskites over the range of more than 100 degrees by modifying the structures of alkylammonium-derived organic cations. Through the introduction of alkyl chain branching and extending the length of the base alkylammonium cation, melting temperatures of as low as 172 °C can be achieved and high quality thin films of layered hybrid lead iodide perovskites can be made using a solvent-free melt process with no additives and in ambient air. Additionally, we show that a similar concept can be translated to the corresponding layered bromides, with slightly higher observed melting temperatures. The design rules established here can guide the discovery of new melt-processable perovskite materials for low-cost high performance devices.

## Abstract 2

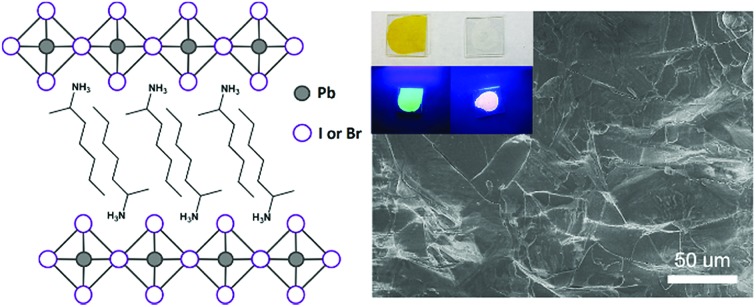
Melting temperature of layered lead halide hybrid perovskites can be tuned by designing branched organic cation structures.

## Introduction

Over the past few years, increasing interest and research efforts have been directed towards hybrid organic–inorganic perovskite (HOIP) materials, particularly lead-halide-based systems, because of their novel electronic and optical properties.^1^ They exhibit suitable band gap energy, strong light absorption, effective carrier separation after excitation and benign defect tolerance, which render them attractive as active layers in photovoltaics and other optoelectronic devices.^2^–^7^ The application of these HOIPs has been expedited because a variety of facile approaches exist to prepare high quality HOIP films for device integration. Solution processing provides versatile pathways to making thin films at relatively low temperature and therefore this approach has been widely adopted and perfected.^8^–^11^ So far, the power conversion efficiencies of solar cells fabricated by solution processes (especially spin-coating) have been rapidly increasing, with the current record reaching over 23%.^12^

Compared with solution processing, alternative approaches such as melt processing have been shown to be applicable in processing HOIPs, polymers, inorganic materials and other hybrid materials.^13^–^20^ Melt processing offers several advantages over solvent-based solution methods, including removing the need for solvents (a potential waste product), ability to control crystal orientation using temperature gradients, and one-step encapsulation protection without external adhesives. However, to meet the requirements for melt processing, the target materials should melt congruently, and should be stable around the melting temperature. Additionally, to minimize the energy input for the process, the melting temperature should be reduced to a low value.

Due to the low thermal stability of lead halide perovskites, they typically decompose before melting,^21^ rendering them incompatible with melt processing. Therefore, to enable these materials for melt processing one must lower the melting temperature safely below the decomposition point. Recently we have demonstrated the feasibility of tuning the thermal properties of phenethylammonium (PEA)-based layered lead iodide perovskites *via* structural modification, to achieve effective melt processing of these materials at relatively low temperature in an ambient atmosphere.^22^ Combined with results of Sn-based layered perovskites, we were able to establish some design principles towards synthesizing low melting-temperature layered HOIPs—*e.g.*, in PEA-based layered perovskites, substitutions closer to the ammonium group (*i.e.*, either β- or 2-positions) tend to lower the melting temperature while substitution further away from the ammonium group (3- or 4-position on the phenyl ring) will increase the temperature.^13^,^14^ However, for the lowest melting temperature (β-methyl-PEA)_2_PbI_4_ system, a small amount of decomposition can still be observed and a 10 wt% excess of the corresponding organic iodide salt needs to be added to compensate for losses of the organic component during thermal processing.

To further investigate the applicability of the proposed design rules over a wider range of hybrid structures and to create material candidates that melt at much lower temperature, we extended our effort into the alkylammonium-based layered lead halide perovskites. We find that, by modifying the structure of the alkylammonium cations, for example extending the alkyl chain length or by introducing branching near the ammonium group, it is possible to create target compounds (lead-iodide-based perovskites) with melting temperatures of as low as ∼170 °C, *i.e.*, comparable to that of reported lowest-melting-temperature Sn-based layered perovskites.^14^ Analogous lead bromide systems are found to melt at higher temperature compared to (same organic cation) lead iodide systems. The crystal structures of the new lead-iodide-based materials have also been determined and the possible correlation between octahedral distortion, nitrogen atom penetration and melting transition temperature are discussed. Coupled with differential scanning calorimetry (DSC) data, the detailed phase transitions of the two lowest melting temperature compounds were studied by *in situ* temperature dependent powder X-ray diffraction. Finally, we demonstrate that melt processing of these low melting temperature materials can be easily achieved under ambient conditions for both lead iodide and bromide compounds, and present the optical properties of the melt-processed and corresponding spin-coated films of the targeted compounds. Gaining a better understanding of thermal property tuning *via* structure modification can pave the way for rational design of new functional HOIP materials that can be processed using this versatile melt processing approach.

## Results and discussion

**Fig. 1:**
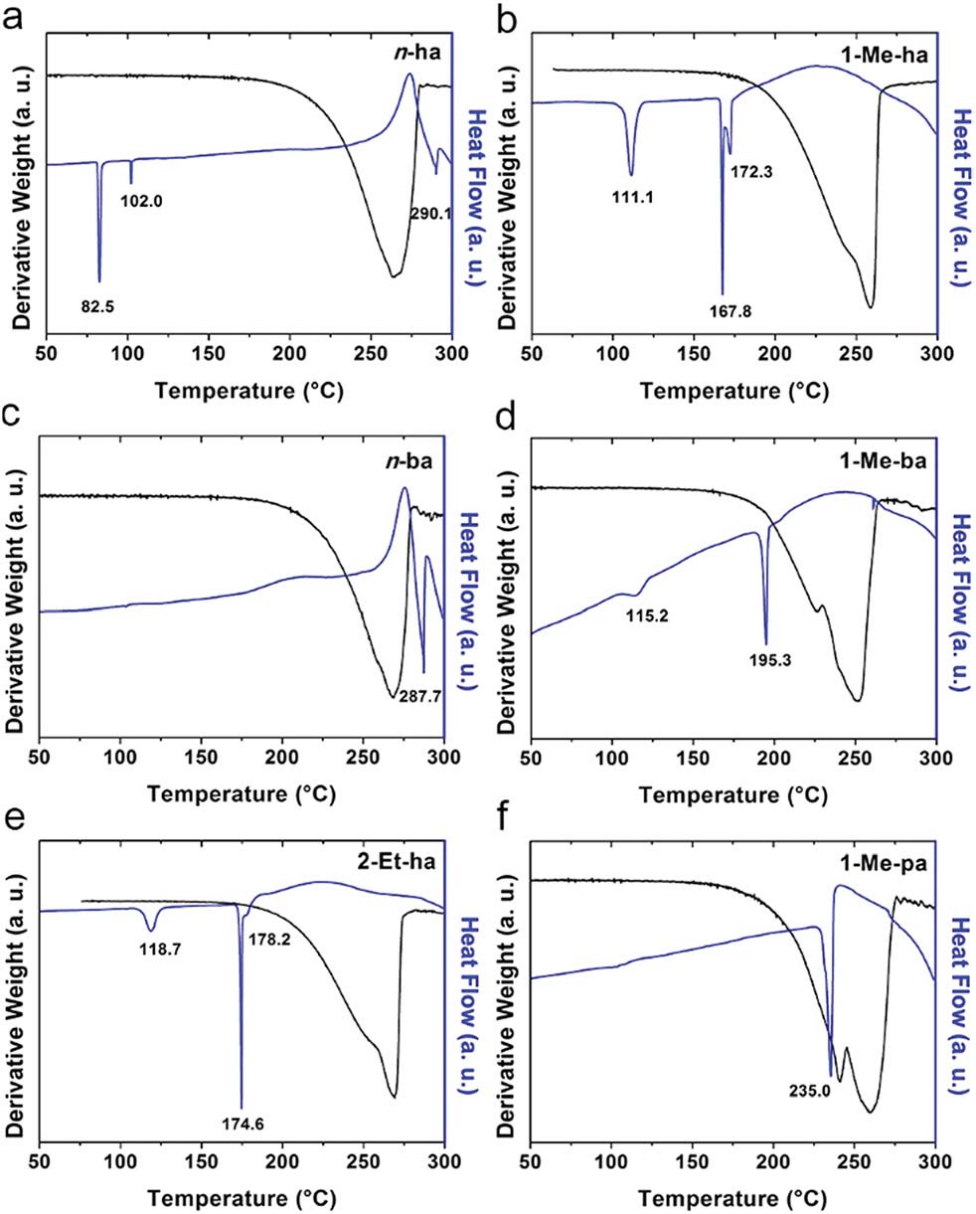
Thermogravimetric analysis (TGA) and differential scanning calorimetry (DSC) scans of (a) (*n*-ha)_2_PbI_4_, (b) (1-Me-ha)_2_PbI_4_, (c) (*n*-ba)_2_PbI_4_, (d) (1-Me-ba)_2_PbI_4_, (e) (2-Et-ha)_2_PbI_4_ and (f) (1-Me-pa)_2_PbI_4_, using a ramp rate of 5 °C min^–1^.

Six alkylammonium cations (*n*-butylammonium (*n*-ba), *sec*-butylammonium (1-Me-pa), 1-methyl-butylammonium (1-Me-ba), *n*-hexylammonium (*n*-ha), 1-methyl-hexylammonium (1-Me-ha), 2-ethyl-hexylammonium (2-Et-ha)) with different chain length and structural motif (linear or branched) were chosen and the corresponding layered lead iodide perovskite compounds were synthesized *via* slow cooling from concentrated hydriodic acid solutions (see ESI† for more detail). The powder X-ray diffraction (XRD) patterns of these compounds (Fig. S1†) show predominantly a series of peaks corresponding to the interlayer spacing between lead iodide sheets. These peaks shift systematically towards smaller two-theta direction as the organic cation chain length increases, clearly indicating a layered nature. These compounds were studied using thermogravimetric analysis (TGA) and differential scanning calorimetry (DSC) (Fig. 1). While they are stable up to ∼190 °C under nitrogen purge, continuous weight loss occurs above 200 °C to ∼350 °C, due to the loss of the organic component, as was also observed for the PEA based compounds. Regardless of the organic cation type (phenethyl- or alkyl-ammonium, with or without substitution or side chain), the decomposition of *n* = 1 layered lead iodide compounds follows a similar pathway, with no clear change in thermal stability noted by changing the organic cation. The DSC curves from room temperature to 300 °C exhibit one or more endothermic peaks and a very broad exothermic feature above 200 °C. The earlier endothermic peaks (if any) are assigned to structural phase transitions and the last endotherm is assigned to the melting transition, as confirmed by visual examination of the solid sample as they melt on a hot plate. The exotherm coincides with the weight loss peak in the derivative weight curve and corresponds to decomposition. The melting transition temperatures extracted from the DSC curves of these compounds (Table 1) range from as low as 172 °C to as high as 290 °C (it is worth noting that, for higher melt-temperature (>250 °C) compounds, melting occurs with some degree of partial decomposition), indicating that organic cation modification provides a highly effective way of tuning the thermal properties of this materials class.

**Table 1:** Phase transition temperatures of layered lead iodide and bromide based perovskite compounds extracted from DSC scans

Organic cation	Cation structure	Structural transition (°C)	Melting transition (°C)
*n*-ba	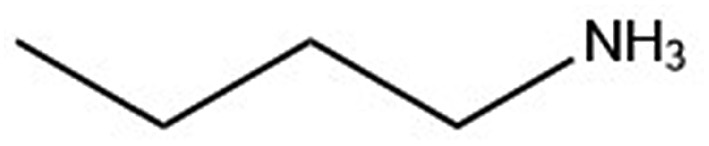	—	287.7
*n*-ha	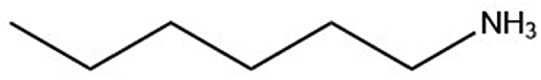	82.5/102.0	290.1
1-Me-pa	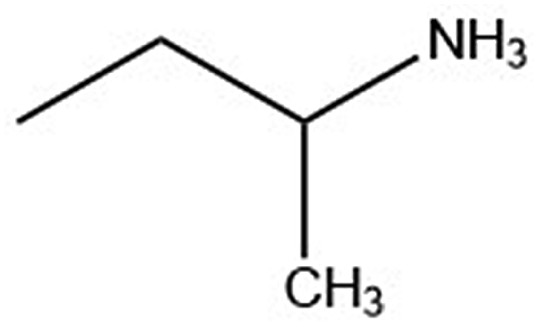	—	235.0
1-Me-ba	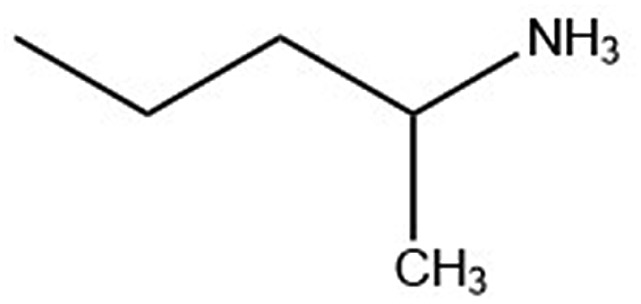	115.2	195.3
2-Et-ha	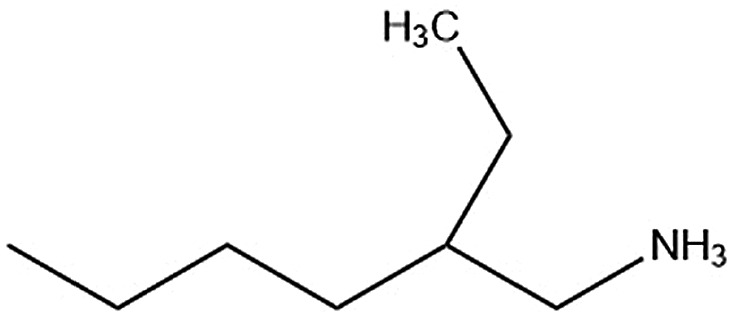	118.7/174.6	178.2
1-Me-ha	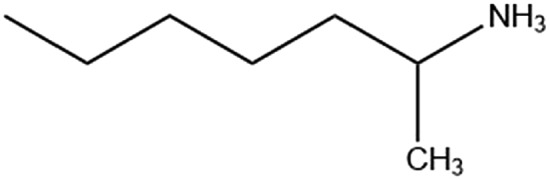	111.1/167.8	172.3
*n*-ha (bromide)	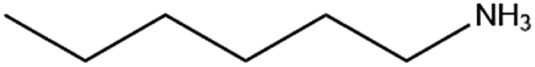	—	—
1-Me-ha (bromide)	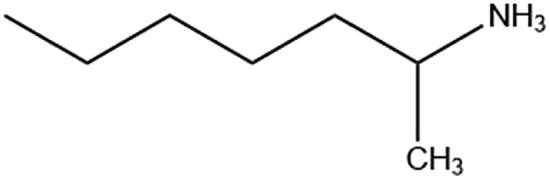	67.1	200.0

Examination of the melting temperatures of these layered perovskites with different organic cation structures reveals two clear trends. First, branching on the alkyl chain near the ammonium group end significantly lowers the melting temperature. For example, *n*-ba and 1-Me-ba based compounds have melting temperature of 287.7 °C and 195.3 °C, respectively, and *n*-ha, 1-Me-ha and 2-Et-ha based compounds have melting temperatures of 290.1 °C, 172.3 °C and 178.2 °C, respectively. Comparing organic cations with the same alkyl chain length, introducing a side chain (methyl or ethyl group substitution) near the ammonium end (at 1- or 2-position) can effectively lower the melting temperature by up to ∼100 °C. Second, for organic cations with a similar structure (*i.e.* side chain at the same position), a longer alkyl chain backbone leads to lower melting temperature—*e.g.*, as the chain length increases from 1-Me-pa, 1-Me-ba to 1-Me-ha, the melting temperature decreases from 235.0 °C to 172.3 °C. These findings here for the alkylammonium cations agree with previous results for PEA-based lead and tin iodides,^14^,^22^ indicating that introducing substitution (branching) near the ammonium group is a general method to lower the melting temperature of layered perovskites. We hypothesize that the variation in the organic cation structure (linear or branched) likely results in different steric impact on the hydrogen bonding interaction between the organic and inorganic components. At the same time, the alkyl chain length can further impact the interaction between neighboring inorganic layers. Knowing this, we can create lead-iodide-based compounds that melt well below decomposition, making them ideal candidates for melt processing.

Recently, layered lead bromide perovskites have been heavily investigated for light emitting applications.^23^–^25^ To both target these functional systems and to test whether the design rules established for the lead iodides translate over to other halides, we sought to extend this melt-processing approach to the bromide systems. The *n*-ha- and 1-Me-ha-based lead bromide compounds were synthesized and characterized (Fig. S2 and S3†). Both compounds present a layered structure, similar to the iodide counterparts.^26^ DSC curves of these two compounds show that (*n*-ha)_2_PbBr_4_ does not exhibit melting behavior below 300 °C, while (1-Me-ha)_2_PbBr_4_ melts at around 200 °C, confirmed by visual examination of the sample heated on a hotplate. These results indicate that the design rule of melting temperature suppression within branched alkylammonium-based cations translates from the iodides to other halide systems. Additionally, bromide systems examined exhibit a higher melting temperature than corresponding iodide-based materials.

**Fig. 2:**
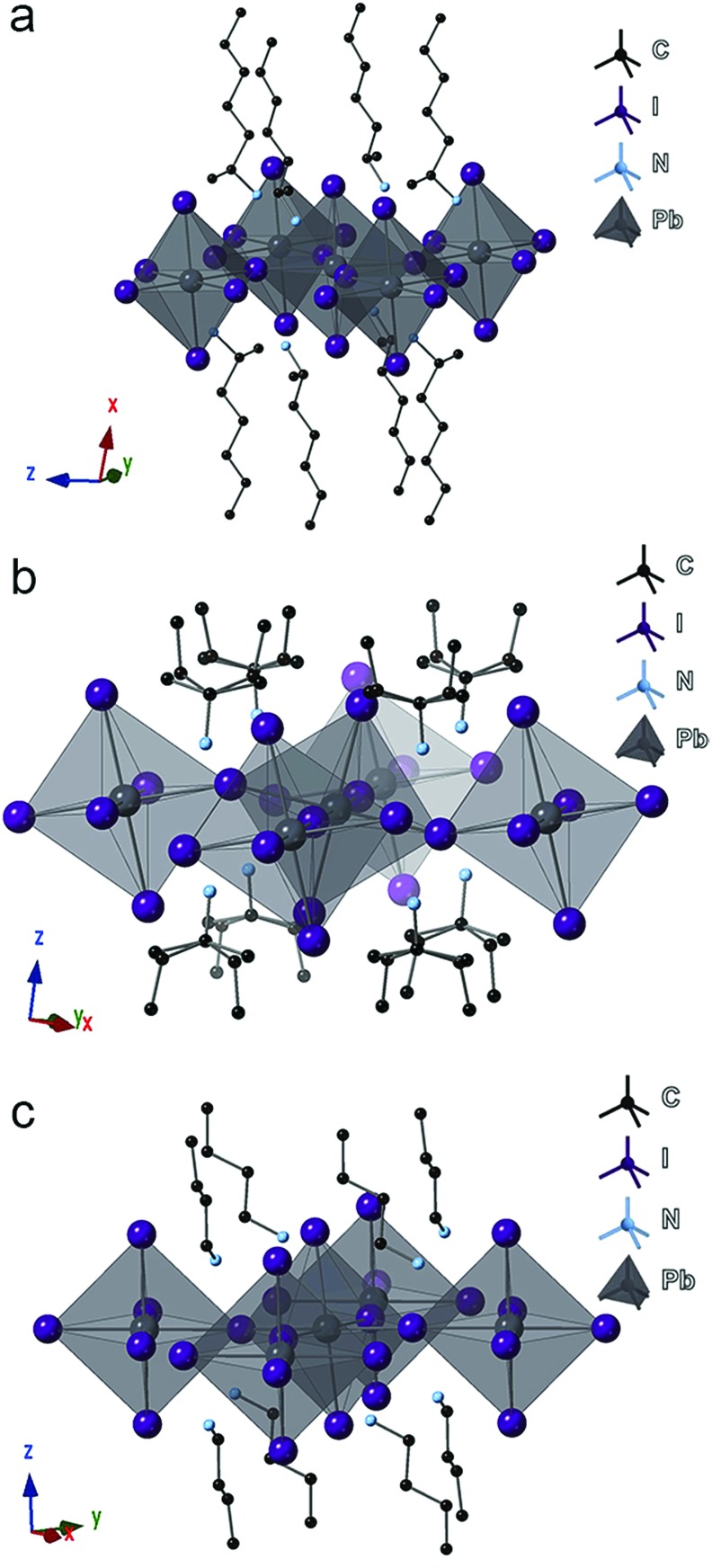
Crystal structure illustrations of (a) (1-Me-ha)_2_PbI_4_, (b) (1-Me-pa)_2_PbI_4_, and (c) (*n*-ba)_2_PbI_4_. Color code: black, Pb; purple, I; light blue, N; dark grey, C; hydrogen atoms are omitted for clarity.

Focusing on the lead-iodide-based systems, single crystal X-ray diffraction (SCXRD) data (Table S1†) were collected at room temperature for the previously unreported four compounds: (2-Et-ha)_2_PbI_4_ (CCDC#; 1863838), (1-Me-ha)_2_PbI_4_ (CCDC#; 1863839), (1-Me-ba)_2_PbI_4_ (CCDC#; 1863836) and (1-Me-pa)_2_PbI_4_ (CCDC#; 1863837) (Table S1 and Fig. S4†). Crystal structures of the two known compounds ((*n*-ha)_2_PbI_4_ and (*n*-ba)_2_PbI_4_) were adopted from previous reports and included for comparison.^27^,^28^ The unit cell parameters of these six compounds are shown in Table 2. Representative structures of (1-Me-ha)_2_PbI_4_, (1-Me-pa)_2_PbI_4_ and (*n*-ba)_2_PbI_4_ are shown in Fig. 2a–c. Structurally, these six compounds consist of anionic corner-sharing Pb–I octahedral layers with interleaving alkylammonium cations. These alkylammonium cations form an alternating organic bilayer, with the ammonium groups (–NH_3_) pointing towards the inorganic layers and residing in the cavity formed by four nearest-neighbor axial iodine atoms. Apart from the similarities noted for these six structures, one interesting feature is that (1-Me-pa)_2_PbI_4_ shows much increased out-of-plane distortion and no in-plane distortion, leading to a puckered inorganic lattice, and such distortion seems to manifest only in the [110] direction within the layer, with one Pb–I–Pb bond angle of ∼158° while the other Pb–I–Pb bond angle is exactly 180° in the [11[combining macron]0] direction (Fig. 2b). The remaining five compounds have both in-plane and much smaller out-of-plane distortions in both *x* and *y* directions. The (1-Me-pa)_2_PbI_4_ compound also exhibits organic cation disorder (two conformations rotated 180° along the N–C bond), while no disorder is observed in the rest of the structures.

**Table 2:** Crystallographic information of the six target lead-iodide-based compounds from single crystal X-ray diffraction

Organic cation	Temperature/K	Space group	Unit cell parameter/Å	Unit cell volume/Å^3^
*n*-ba^27^	293	*Pbca*	*a* = 8.8764(1)	2129.67(5)
			*b* = 8.6925(1)	
			*c* = 27.6014(5)	
*n*-ha^27^	293	*Pbca*	*a* = 8.9413(2)	2540.24(11)
			*b* = 8.6874(2)	
			*c* = 32.7027(10)	
1-Me-pa	293	*P*4_2_/*ncm*	*a* = 8.9353(2)	1993.10(15)
			*b* = 8.9353(2)	
			*c* = 24.9636(13)	
1-Me-ba	296	*P*2_1_/*c*	*a* = 15.0424(8)	1144.84(10)
			*b* = 9.2398(4)	
			*c* = 8.5456(4)	
			*β* = 106.155(3)°	
2-Et-ha	296	*P*2_1_/*c*	*a* = 18.6958(7)	1456.53(9)
			*b* = 8.8497(3)	
			*c* = 8.8421(3)	
			*β* = 95.3669(12)°	
1-Me-ha	296	*P*2_1_/*c*	*a* = 17.4586(5)	1350.06(8)
			*b* = 9.2513(3)	
			*c* = 8.5864(3)	
			*β* = 103.2228(10)°	

To quantify and analyze the octahedral distortions within the six perovskite compounds, we define such distortions in terms of the distortion of the PbI_6_ octahedra and the tilting of the octahedra in and out of the inorganic stacking plane (see definitions of the parameters in ESI and Fig. S5†).^29^–^31^ The values of these distortion parameters are summarized in Table S2.† It has been hypothesized that subtle changes in the crystal structure (*e.g.* cation penetration or octahedral tilting) can lead to changes in optical and thermal properties.^32^–^35^ For the six target compounds of the current study, for structures with the same carbon chain length—*e.g.*, (1-Me-ha)_2_PbI_4_, (2-Et-ha)_2_PbI_4_, and (*n*-ha)_2_PbI_4_—the corresponding organic cation penetration depths are 0.528 Å, 0.547 Å and 0.626 Å, and the out-of-plane tilting angles are 4.75°, 4.87° and 5.85°, respectively. With methyl or ethyl substitutions close to the N end of the cation, the increased steric effect of this substitution appears to correlate with smaller cation penetration into the inorganic framework, which in turn results in reduced out-of-plane octahedral distortion. A plot of the penetration depth and out-of-plane values from the broader family of hybrid layered lead iodide perovskites in this paper and from previous literature is constructed (Fig. S6†).^29^,^36^–^38^ From this plot it becomes apparent that there is not a broadly-applicable well-behaved correlation between penetration depth and out-of-plane distortion. Clearly different organic ammonium cation types (aliphatic or aromatic, functionalized or unfunctionalized) can lead to distinctive degrees of distortion and independent trends.

Melting of the lead-iodide-based HOIPs transforms them into a clear yellow liquid and requires overcoming the interaction between inorganic and organic components (as well as covalent/ionic bonding interaction within the Pb–I framework). The strength of these interactions determines how easily the compound melts and we therefore try to establish some correlation between the octahedral distortion, hydrogen bonding interaction and the melting temperature of the corresponding compounds (shown in Fig. S7†). The penetration depth or the average hydrogen bonding distances (that is, three distances between closest H and I atoms involved with hydrogen bonding) does not show obvious direct correlation with melting temperature. It is also obvious that the two data points from the (1-Me-pa)_2_PbI_4_ structure are significantly larger than corresponding values for the other five compounds, likely due to the structural differences and larger out-of-plane distortion mentioned previously. However, there seems to be a strong correlation between the penetration depth and the hydrogen bonding strength, for all data points including the outlier. It is worth noting that, since these compounds typically undergo structural phase transitions before melting (*e.g.*, see Fig. 1 and Table 1), the structural characteristics described above may not hold at higher temperature. Therefore, correlations between structure and melting temperature would need to take into account the relevant structures present at the higher temperatures preceding melting.

**Fig. 3:**
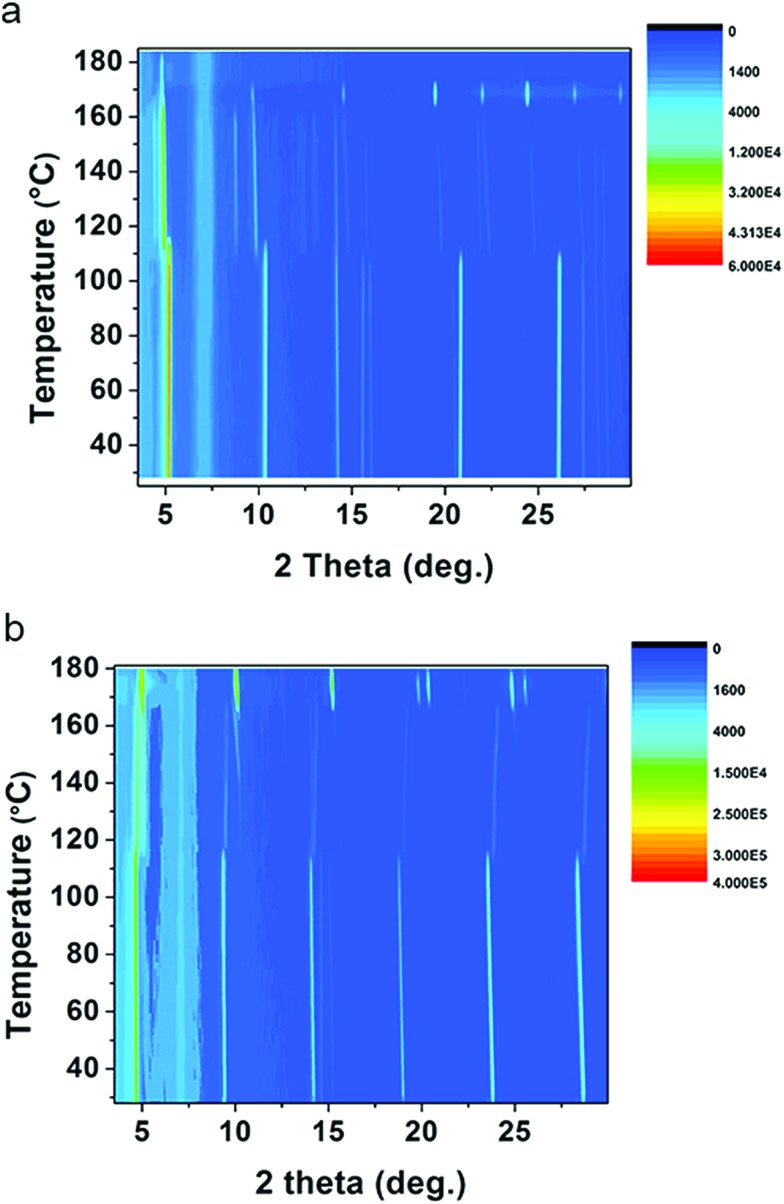
*In situ* temperature-dependent powder XRD measurements of (a) (1-Me-ha)_2_PbI_4_ and (b) (2-Et-ha)_2_PbI_4_, using CuKα radiation (color scale: signal counts).

As discussed above, (1-Me-ha)_2_PbI_4_ and (2-Et-ha)_2_PbI_4_ have melting temperature below 180 °C, making them particularly interesting for melt processing. For both of these compounds, the DSC curves show two phase transitions in a similar temperature range before melting: the first phase transition between 110 °C and 120 °C and the second above 165 °C, immediately prior to melting. Temperature dependent *in situ* powder X-ray diffraction was performed to carefully study these phase transitions (Fig. 3). The samples were ramped to 180 °C from room temperature and scans were repeatedly taken over this temperature range, using a two theta scan range from 3° to 30°. For (1-Me-ha)_2_PbI_4_, from room temperature to ∼110 °C, the structure undergoes a very small lattice contraction in the interlayer direction, as the more intense (*h*00) peaks at approximately 5.2°, 10.3°, 15.6°, 20.9° and 26.0° gradually shift towards higher angle with increasing temperature. The first phase transition occurs at ∼110 °C, as evident from the emergence of new peaks at around 4.4°, 8.8°, 14.8°, 19.8° and between 21°–25°, as well as the abrupt shift for the ∼10° peak and the peak disappearance at ∼20.8° and ∼26°. The lattice then undergoes expansion with increasing temperature as all the peaks shift to lower angle. The second phase transition occurs at ∼163 °C, with sudden disappearance of several peaks and peak intensity change. The sample eventually melts above 170 °C and shows no sharp diffraction peaks, indicating an amorphous liquid state. Similarly, (2-Et-ha)_2_PbI_4_ shows no phase transition, but thermal expansion until ∼114 °C. After the first phase transition, it undergoes thermal contraction in the interlayer direction as the (*h*00) peaks shift towards higher angle with increasing temperature. Interestingly, the peak at around 10° (indexed as the (110) peak, based on unit cell parameters from SCXRD) seems to shift to smaller angle, indicating an expansion in the in-plane direction. The second phase transition occurs at ∼170 °C, with sudden peak shift and intensity change. It melts above 180 °C and the peaks start to fade into the background. Overall, the phase transition information from the temperature-dependent XRD data agrees well with the DSC data, and the small differences between the phase transition temperatures in DSC and *in situ* XRD most likely arise due to the inherent error of extracting transition temperatures from *in situ* XRD measurement, which employs an average of the temperature during each five-minute scan window. The changes of diffraction patterns during the temperature ramp-up can shed light on the structural changes that eventually lead to melting. No peaks from PbI_2_, which is a common decomposition product, are observed, and a series of (*h*00) reflections remain clearly visible throughout the measurement, but have small two-theta shift with temperature. This suggests that the 2D nature of the Pb–I inorganic layer remains intact and the organic component nominally stays in between the layers before melting, even under continuous nitrogen purge.

**Fig. 4:**
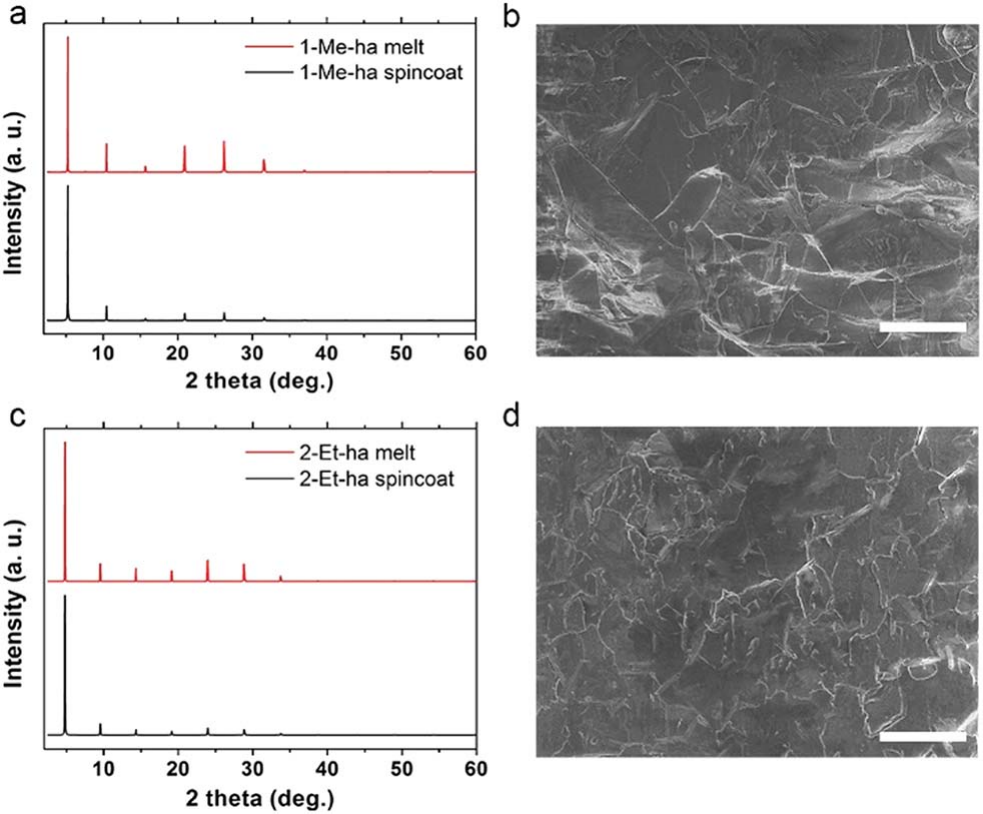
Comparisons of XRD patterns of spin-coated and melt-processed films and SEM images of the melt-processed films of (1-Me-ha)_2_PbI_4_ (a and b) and (2-Et-ha)_2_PbI_4_ (c and d). Scale bar: 50 μm.

Through the above studies, modification of the organic cation structure has been shown to successfully tune the thermal properties of layered lead iodide perovskites, yielding compounds (1-Me-ha)_2_PbI_4_ and (2-Et-ha)_2_PbI_4_ with record-low melting temperatures. Compared with PEA-based lead iodide systems, because these two compounds melt well before decomposition, no additional organic iodide salt needs to be added during the melt process, and melt processing of phase pure, highly crystalline films can readily be achieved under ambient atmosphere conditions (Fig. S8†). The solid is melted on the glass substrate with an 8-micrometer-thick Kapton film cover. The melt liquid is then pressed from the top to form a thin liquid layer between the substrate and the Kapton film. A solid film is obtained by solidification of the liquid layer after removing from the heat source. The XRD of the melt-processed films exhibit predominantly a series of (*h*00) narrow peaks, due to the 2D nature of these materials (Fig. 4a and c). No impurity (*e.g.* PbI_2_ or other related low-dimensional phases^39^,^40^) can be observed. The peak positions are essentially the same and the relative intensities of the melt-processed and spin-coated films show only very minor differences, likely due to changes in degree of preferred orientation and/or crystallinity. Examination of the melt-processed films using scanning electron microscopy (SEM) shows large lateral grain size of more than 30 μm, consistent with the narrow (*h*00) peaks in the XRD, and good pinhole-free film coverage (Fig. 4b and d). The thickness of the above-mentioned films is estimated to be around 500–600 nm by cross sectional SEM, and can be further tuned by changing the pressure applied during the melt process.

**Fig. 5:**
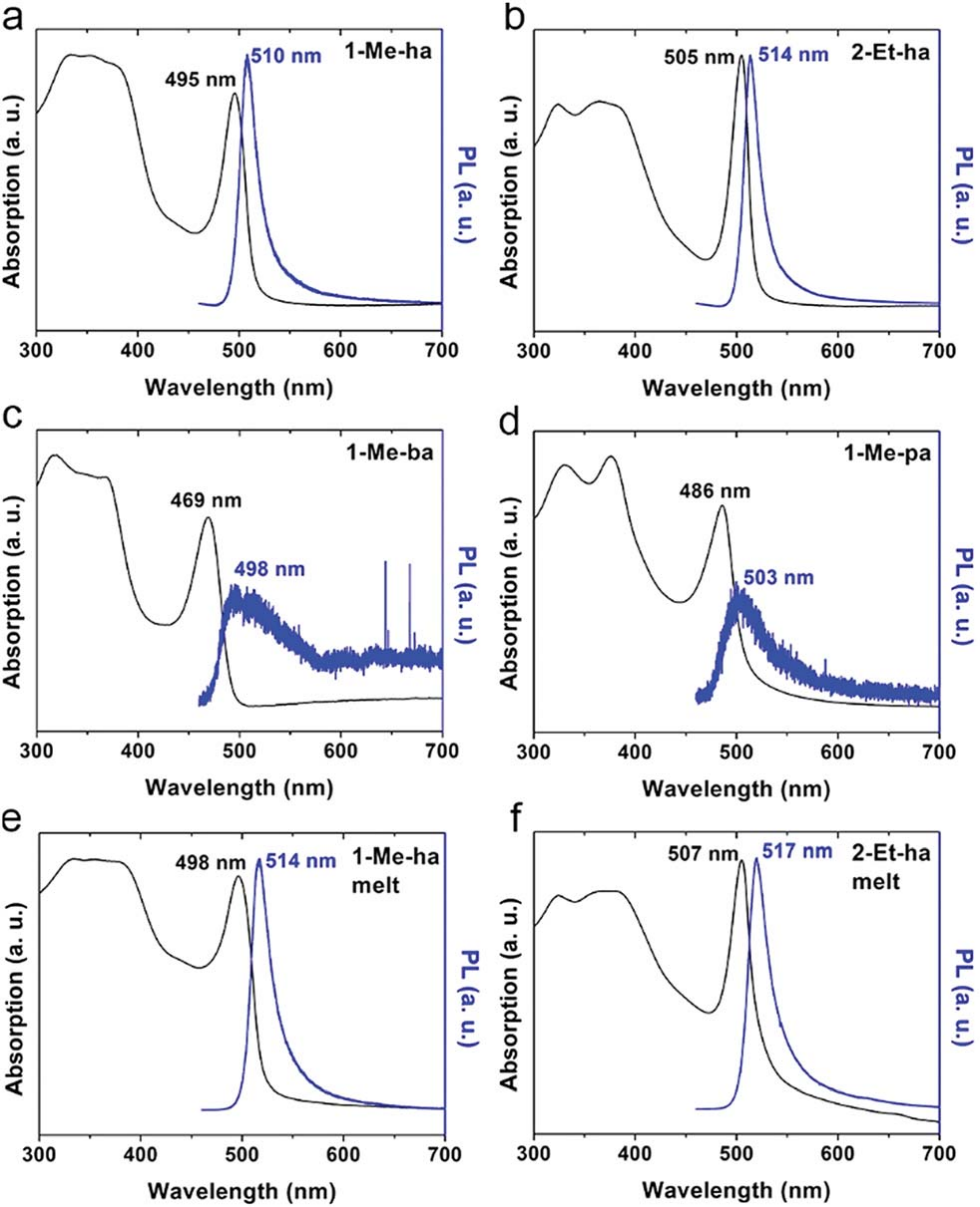
UV-vis absorption and photoluminescence (442 nm excitation) spectra of spin-coated film of (a) (1-Me-ha)_2_PbI_4_, (b) (2-Et-ha)_2_PbI_4_, (c) (1-Me-ba)_2_PbI_4_ and (d) (1-Me-pa)_2_PbI_4_ and melt-processed films of (e) (1-Me-ha)_2_PbI_4_, (f) (2-Et-ha)_2_PbI_4_.

The optical properties of the four new lead-iodide-based compounds were studied using ultraviolet-visible (UV-vis) light absorption and photoluminescence (PL) measurements. Fig. 5 shows the absorption and PL spectra of spin-coated films of (1-Me-ha)_2_PbI_4_, (2-Et-ha)_2_PbI_4_, (1-Me-ba)_2_PbI_4_ and (1-Me-pa)_2_PbI_4_. A strong exciton absorption peak can be observed for all four compounds, with the peak position at 2.51 eV (495 nm), 2.46 eV (505 nm), 2.64 eV (469 nm), 2.55 eV (486 nm), respectively. The typical band gap energy of these *n* = 1 layered lead iodide perovskites is between 2.5–2.6 eV;^38^ however, because of the broadening of the exciton peak at room temperature, the overlap between the exciton peak and the band edge absorption hinders the direct extraction of the band gap energy at room temperature. Both (1-Me-ha)_2_PbI_4_ and (2-Et-ha)_2_PbI_4_ films show intense PL emissions with narrow PL peaks centered at 2.43 eV (510 nm) and 2.41 eV (514 nm), respectively. On the other hand, (1-Me-ba)_2_PbI_4_ and (1-Me-pa)_2_PbI_4_ have very weak PL emissions at around 2.46 eV that are barely detectable even at 100% excitation laser power. Compared to the spin-coated films, melt-processed films of (1-Me-ha)_2_PbI_4_ and (2-Et-ha)_2_PbI_4_ have similar absorption and PL spectra, with the exciton and PL peaks red shifted by about 0.02 eV and, in both cases, slightly broader peak profiles (Fig. 5e and f). These minor changes are likely due to a slight increase in defects near the band edge, originating from organic cation or iodide loss.^41^,^42^ The increase in absorption close to the band edge (more overlap between the exciton peak and band edge) of the melt-processed films could also be due to the increased scattering from rough film surfaces (due to removal of the Kapton film cover) and film thickness differences.^43^–^45^ Melt-processed (1-Me-ha)_2_PbBr_4_ films show an absorption onset at ∼400 nm and broadband white PL emission (Fig. S9 and S10†), which is similar to what has been reported in other lead bromide layered perovskites.^46^ Translation of the melt-processing approach to the lead bromide perovskites further broadens the scope of melt processing within hybrid perovskites and potential application.

## Conclusions

In conclusion, the thermal properties (*i.e.* melting temperature) of both Sn- and Pb-based layered perovskite compounds can be tuned with organic cation structural modification. In this work, several alkylammonium-based layered lead halide perovskite compounds have been synthesized, structurally determined and optically characterized. The lead bromide perovskites are found to melt at higher temperature than the iodides. The correlation between the organic cation structure and the melting temperature suppression is considered—*i.e.*, branching near the ammonium group and longer alkyl chain length effectively decreases the melting temperature by 100 °C, down to ∼170 °C and well below the decomposition temperature. This provides a comfortable temperature window for solvent-free melt processing, in ambient air, of lead halide HOIP thin films with minimal energy input. As a result, we show that these melt-processed films show high crystallinity and phase purity, with essentially the same optical properties as the spin coated analogues. Furthermore, the findings here can be formulated into design rules that will facilitate the discovery of other new low melting temperature hybrid perovskite compounds (not just lead halide based perovskites) that can be readily melt-processed into high quality thin films and that also possess potentially interesting properties, such as relatively low band gap for photovoltaic and optoelectronic applications, light emission or magnetic behavior for spintronics.^47^–^49^

## Conflicts of interest

There are no conflicts to declare.

## Supplementary Material

Supplementary information

Crystal structure data
