# Supplementary material for: Melting temperature suppression of layered hybrid lead halide perovskites via organic ammonium cation branching
Source: Chem Sci. 2018 Nov 9;10(4):1168–75. doi: 10.1039/c8sc03863e (PMC6349064; doi:10.1039/c8sc03863e)
Supplement: Supplementary file 1 [file SC-010-C8SC03863E-s001.pdf]

Electronic Supplementary Material (ESI) for Chemical Science.
This journal is © The Royal Society of Chemistry 2018

Supporting Information

Melting Temperature Suppression of Layered Hybrid Lead Halide Perovskites via Organic Ammonium Cation Branching

Tianyang Li,[a] Wiley A. Dunlap-Shohl,[a] Eric W. Reinheimer,[c] Pierre Le Magueres[c] and David B. Mitzi[a,b]

[a] Department of Mechanical Engineering and Materials Science, Duke University, Durham, North Carolina 27708, United States

[b] Department of Chemistry, Duke University, Durham, North Carolina 27708, United States

[c.] Rigaku Americas Corporation, 9009 New Tails Drive, The Woodland, Texas 77381, United States

Experimental section

Chemicals.

2-Ethyl-1-hexylamine (2-Et-ha, 98%), 2-Aminoheptane (1-Me-ha, 99%), 2-Aminopentane (1-Me-ba, 97%), *sec*-butylamine (1-Me-pa, 99%), hexylamine (ha, 99%), butylamine (ba, 99.5%), hydrobromic acid (48 wt%) and hydriodic acid (HI) solution (57 wt%, stabilized, 99.95%) were purchased from Sigma Aldrich and used without further purification.

Synthesis.

Alkylammonium iodide salts (i.e. 1-Me-ha·HI, 2-Et-ha·HI, 1-Me-ba·HI and 1-Me-pa·HI) were synthesized by adding stoichiometric amount of HI solution dropwise into corresponding pure alkylamine liquid in a cold water bath, giving a light yellow liquid. After evaporating water while on a hotplate at 150 ˚C, the resulting gel was further vacuum dried at 150 ˚C at 40 mtorr for a week.

In a typical synthesis of 2D lead iodide perovskite crystals, stoichiometric amounts of PbI_2 (0.25 mmol) and the corresponding alkylammonium iodide (0.5 mmol) were added into 0.5 mL HI solution and 0.5 mL methanol. Slow evaporation of methanol over a week yields thin-plate-like crystals. The crystals were filtered and then washed using ethyl ether.

$(n\text{-ha})_2PbBr_4$ was synthesized by cooling of a 0.125M precursor solution (using aqueous 48 wt% HBr as solvent) from 90 ˚C to room temperature over 48 hours. The crystals were filtered and then washed using ethyl ether. $(1\text{-Me-ha})_2PbBr_4$ was synthesized using a similar process to the iodide compounds, in which 0.125 mmol $PbBr_2$ and 0.25 mmol of 1-Me-ha were added into 1.0 mL HBr solution and 0.5 mL methanol. Slow evaporation of methanol over a week yields a white flaky solid, which was filtered and then washed using ethyl ether.

Spin coated film preparation.

The spin-coated films were prepared by dissolving corresponding perovskite crystals in DMF to form a 0.5 M precursor solution, followed by spin coating the solution onto glass substrates at the speed of 5000 rpm for 30s, and annealing afterwards at 100 ˚C for 5 min in a nitrogen-filled glovebox.

Melt processed film preparation.

0.5 mg of the perovskite powder was placed onto a 1.2 mm thick glass substrate and then covered with a piece of 8-µm-thick Kapton sheet. The substrate was then placed onto a preheated hot plate (temperature set to 5 ˚C higher than the melting point) and pressed on top with another preheated glass

substrate. After the solid melts and forms a yellow liquid, the substrate was then removed from the hot plate and cooled down naturally.

Characterization methods.

Thermogravimetric analysis (TGA) measurements were performed on a TA Q50 instrument at a ramp rate of 5 ˚C/min from 25 ˚C to 400 ˚C, under nitrogen gas flow, and with a 4 mg sample size. Differential scanning calorimetry measurements were performed using a TA Discovery DSC instrument at a ramp rate of 5 ˚C/min from 25 ˚C to 300 ˚C, using a hermetically-sealed aluminium pan and lid with a 1 mg sample loading. DSC curves of $(ha)_2PbBr_4$ and $(1\text{-Me-ha})_2PbBr_4$ were measured using a TA SDT550 instrument at a ramp rate of 5 ˚C/min from 25 ˚C to 300 ˚C, using a hermetically-sealed aluminium pan and lid with around 3 mg sample loading.

Powder and film X-ray diffraction (XRD) measurements were carried out on a PANalytical Empyrean Powder X-ray diffractometer using CuKα radiation, with X-ray tube operating conditions at 45 kV and 40 mA. Temperature dependent *in-situ* XRD measurements were performed on a PANalytical Empyrean Powder X-ray diffractometer equipped with an XRK 900 reactor chamber using CuKα radiation. Sample powder was loaded onto a ceramic holder and was scanned from 3 to 30 degrees 2θ and ramped from room temperature to 180 °C at 5 °C/min under nitrogen atmosphere.

Morphologies of the spin coated and melt processed films were imaged with a scanning electron microscope (SEM, FEI XL30 SEM-FEG) using a 5 kV accelerating voltage. Optical absorption measurements were performed on a Shimadzu UV-3600 spectrophotometer. Photoluminescence measurements were performed on a Horiba Jobin Yvon LabRam ARAMIS system using a 442 nm laser as excitation.

Single crystal diffraction data of $(1\text{-Me-pa})_2PbI_4$ were collected on a Rigaku XtaLAB Synergy-S instrument and $(1\text{-Me-ha})_2PbI_4$, $(2\text{-Et-ha})_2PbI_4$ and $(1\text{-Me-ba})_2PbI_4$ were collected on a Bruker D8 ADVANCE Series II instrument, all at room temperature using Mo Kα radiation (= 0.71073 Å). The crystal structures were solved and refined using the Olex2 program[1] and Shelx software package[2]. For the $(2\text{-Et-ha})_2PbI_4$ structure, restraint DFIX was used for C-C bond distances and EADP was applied to the three carbon atoms at the end of the alkyl chain to give reasonable isotropic thermal displacement parameters. For the $(1\text{-Me-pa})_2PbI_4$ structure, disorder within the organic cation was observed. For the organic cation, parts 1 and 2 of the disorder model were defined as the methyl group and the longer chain alkyl group, respectively, and set to an occupancy ratio of 50:50 with respect to one another. Through the application of a mirror plane, which passed through the N-C bond of the cation, its other half was generated. For the $(1\text{-Me-pa})_2PbI_4$ structure, as a means to achieve suitable bond distances between singly-bonded carbon atoms within the cation, the restraints DFIX and SADI were used. Reasonable thermal parameters for the atoms in the cation were obtained using the SIMU, RIGU and EADP constraints and restraints. The refinement of the room temperature data leads to a relatively large R_1 value of 0.061 and a large wR_2 value of 0.22. Removing the constraints and restraints will not improve the R values of the refinement and, on the other hand, it will lead to destabilization of the structure, especially the carbon atoms.

Checkcif A level alerts for $(1\text{-Mepa})_2PbI_4$:

```
"_vrf_SHFSU01_1me-pa_roomt_feb
; PROBLEM: The absolute value of parameter shift to su ratio > 0.20
;
_vrf_PLAT080_1me-pa_roomt_feb
; PROBLEM: Maximum Shift/Error ........................... 1.03 Why ?"
```

These two alerts are due to the restraints and constraints applied to the model, which lead to the small degree of rotation of the C-C bond at the end of the alkyl-chain. Due to the rotation and failure to fully converge, the position of the hydrogen atoms on the said carbon atoms cannot be completely settled.

```
"_vrf_PLAT310_1me-pa_roomt_feb
; PROBLEM: H3A Deleted (Close to H3B ) Dist ... 0.148 Ang."
```

This alert is due to the disorder model, where part 1 and part 2 of the 1-Mepa cation are mirrored through a plane that passes through the C-N bond. H3A and H3B belong to different parts of the disorder model.

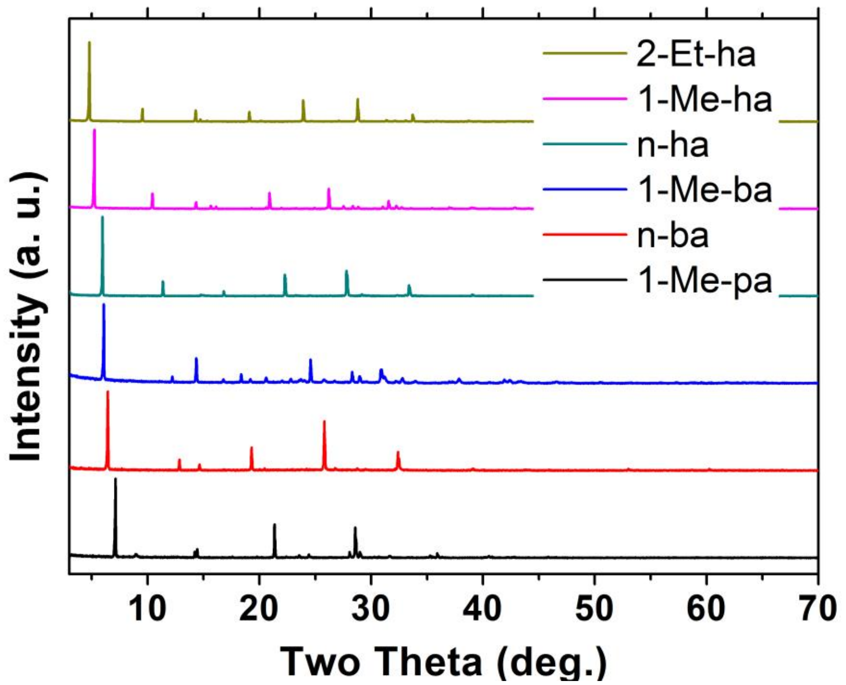

Fig. S1 Powder X-ray diffraction data of six layered lead iodide perovskite compounds with different organic cation structures.

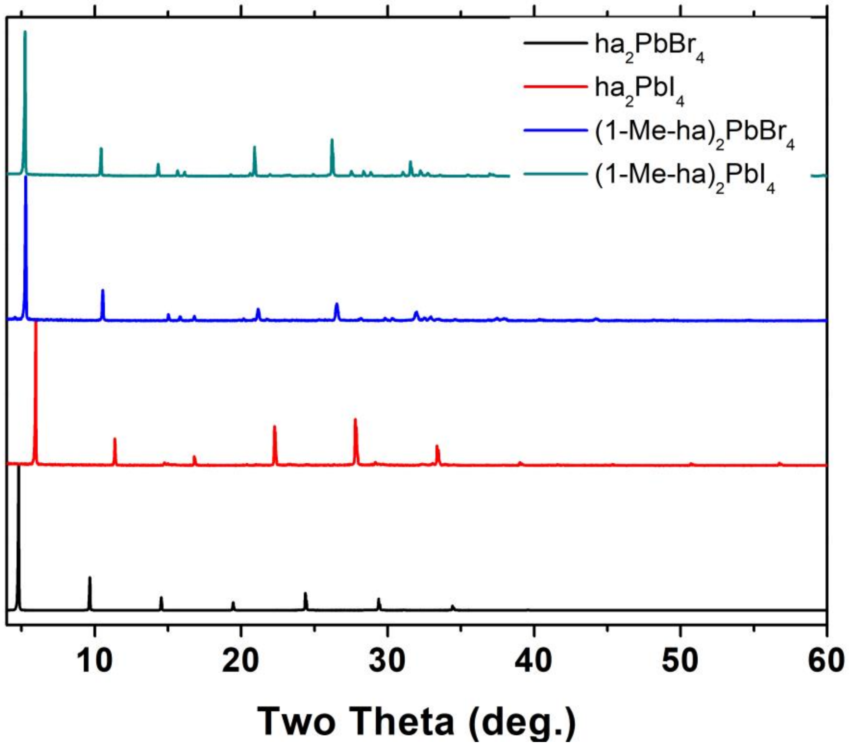

Fig. S2 Powder X-ray diffraction patterns of ha and 1-Me-ha based iodide and bromide compounds. The 1-Me-ha based bromide and iodide compounds show very similar interlayer distances. The pattern of ha_2PbBr_4 matches well with previous reports.[3]

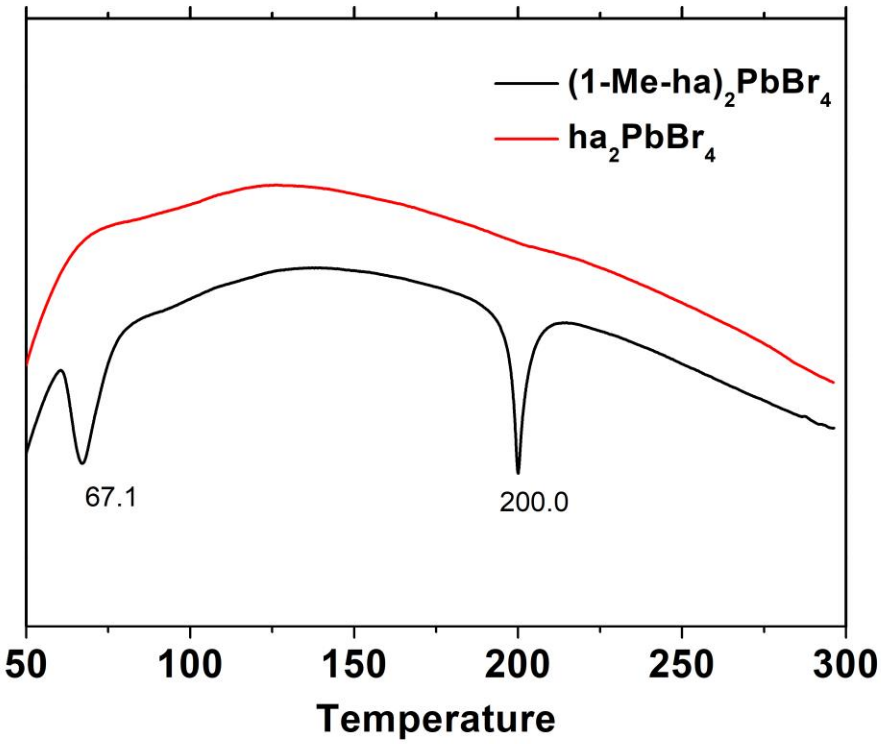

Fig. S3 DSC curves of $(ha)_2PbBr_4$ and $(1\text{-Me-ha})_2PbBr_4$.

Table S1 Summary of crystallographic data of four newly synthesized lead-iodide-based compounds

Compound name	$(1\text{-Me-ha})_2PbI_4$	$(2\text{-Et-ha})_2PbI_4$	$(1\text{-Me-pa})_2PbI_4$	$(1\text{-Me-ba})_2PbI_4$
Empirical formula	$C_{14}H_{36}N_2I_4Pb$	$C_{16}H_{40}N_2I_4Pb$	$C_8H_{24}N_2I_4Pb$	$C_{10}H_{30}$ N_2I_4Pb
Formula weight	947.24	975.28	863.08	891.13
Crystal size (mm)	0.037×0.159×0.171	0.039×0.146×0.185	0.025×0.173×0.321	0.084×0.0.89×0.101
Space group	$P2_1/c$	$P2_1/c$	$P4_2/ncm$	$P2_1/c$
T / K	296	296	293	296
λ / Å	0.71073	0.71073	0.71073	0.71073
a / Å	17.4586(5)	18.6958(7)	8.9353(2)	15.0424(8)
b / Å	9.2513(3)	8.8497(3)	8.9353(2)	9.2398(4)
c / Å	8.5864(3)	8.8421(3)	24.9636(13)	8.5756(5)
α / °	90	90	90	90
β / °	103.2228(10)	95.3669(12)	90	106.155(3)
γ / °	90	90	90	90
V / $Å^3$	1350.06(8)	1456.53(9)	1993.10(15)	1144.84(10)
Z	2	2	4	2
Density / $g·cm^{-3}$	2.330	2.224	2.876	2.585
μ / mm^{-1}	21.635	10.033	14.644	12.751
F(000)	1569	888	1520	792
2*ϑ* for data collection	6.51-53.24	6.35-53.36	5.608-50.05	7.16-52.86
Measured refls.	28742	22885	11131	13347
Independent refls.	2802	3031	972	2354
Observed reflection ($I > 2σ(I)$)	2447	2512	905	2125
Peak and hole / e $Å^{-3}$	1.66/-2.36	1.654/-1.351	2.744/-2.086	0.8/-1.0
R_{int}	0.0356	0.0397	0.0260	0.0216
No. of parameters	100	97	38	82
GOF	1.154	1.028	1.160	1.271
$^aR_1$, wR_2 [$I > 2σ(I)$]	0.0226, 0.0594	0.0288, 0.0705	0.0612, 0.2229	0.0314, 0.0714
$^aR_1$, wR_2 (all data)	0.0283, 0.0621	0.0411, 0.0756	0.0630, 0.2264	0.0360, 0.0724

$^aR_1 = \sum\left||Fo| - |Fc|\right|/\sum|Fo|$, $wR_2 = \{\sum w[(Fo)^2 - (Fc)^2]^2 / \sum w[(Fo)^2]^2\}^{1/2}$

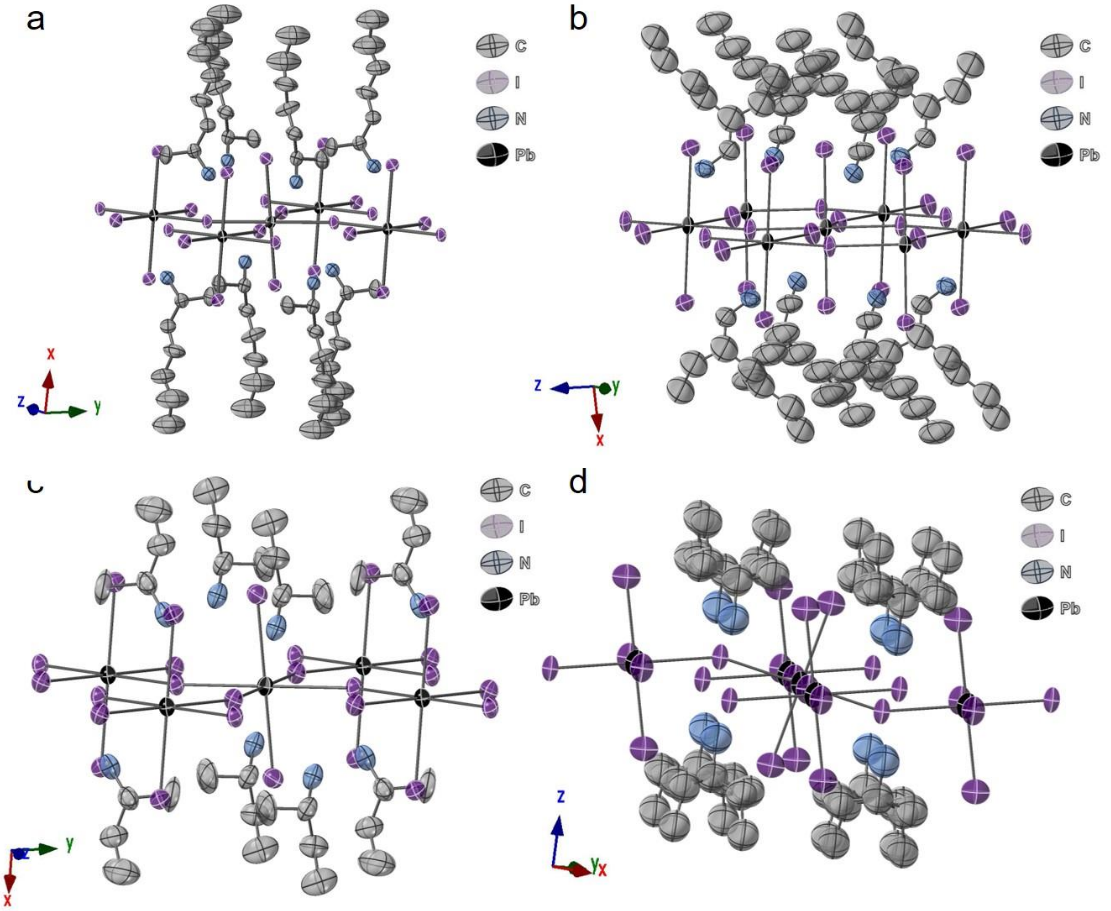

Fig. S4 Crystal structure illustrations of (a) $(1\text{-Me-ha})_2PbI_4$, (b) $(2\text{-Et-ha})_2PbI_4$, (c) $(1\text{-Me-ba})_2PbI_4$ and (d) $(1\text{-Me-pa})_2PbI_4$. Hydrogen atoms are omitted for clarity.

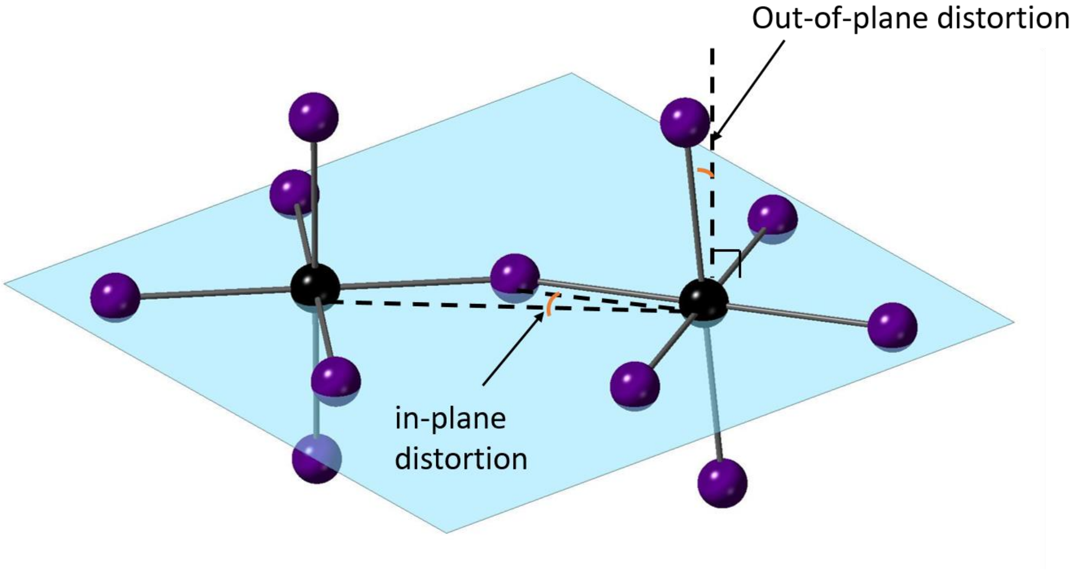

Fig. S5 Scheme of the octahedral in-plane and out-of-plane distortion angles.

Table S2 Summary of structural distortion parameters for the six compounds discussed in this work

	(1-Me-ha)$_2$PbI$_4$	(2-Et-ha)$_2$PbI$_4$	(1-Me-ba)$_2$PbI$_4$	(1-Me-pa)$_2$PbI$_4$	(*n*-ha)$_2$PbI$_4$	(*n*-ba)$_2$PbI$_4$
Octahedral bond distortion *Δd*	7.94E-05	1.06E-05	7.65E-05	7.97E-05	0.799E-05	1.12E-05
Quadratic elongation λ	1.00257	1.00168	1.00298	1.00047	1.000210	1.00148
Pb-I-Pb bond angle / °	153.87	153.98	153.90	158.16/180	155.65	155.08
Out-of-plane/in-plane tilting angle / °	4.75/13.03	4.87/12.97	4.35/13.01	13.44/0	5.85/12.01	5.78/12.29
Cation penetration / Å	0.528	0.547	0.489	0.863	0.626	0.589

The octahedral bond distortion is defined as follows:

$$\Delta \boldsymbol{d} = \frac{1}{6}\sum_i^6 \left(\frac{d_i - d_{\text{avg}}}{d_{avg}}\right)^2 \quad ,$$

where $\Delta \boldsymbol{d}$ defines the octahedral bond distortion, d_i is each Pb-I bond length, and d_{avg} is the average of the six Pb-I bond lengths.

The quadratic elongation is defined as follows:

$$\boldsymbol{\lambda} = \frac{1}{6}\sum_i^6 \left(\frac{d_i}{d_0}\right)^2 \quad ,$$

where d_i is each Pb-I bond length within an octahedron, and d_0 is the bond length for an undistorted octahedron with the same volume.

The in-plane distortion angle is defined as the angle between the projected bridging Pb-I bond and the neighboring Pb-Pb connection within the inorganic stacking plane.

The out-of-plane distortion angle is defined as the angle between the axial Pb-I bond and the direction perpendicular to the inorganic stacking plane. For the ideal (undistorted) layered perovskite structure, both angles should be 0° and, the larger the angle, the stronger the distortion of the perovskite layer away from the ideal structure.

The organic cation penetration, defined as the perpendicular distance between the nitrogen atom and the plane of the axial iodine atoms of the inorganic layer, is another parameter often associated with distortion. In these alkylammonium systems, the penetrations are all positive numbers, meaning that the nitrogen atom falls below the plane of the axial iodine atoms.

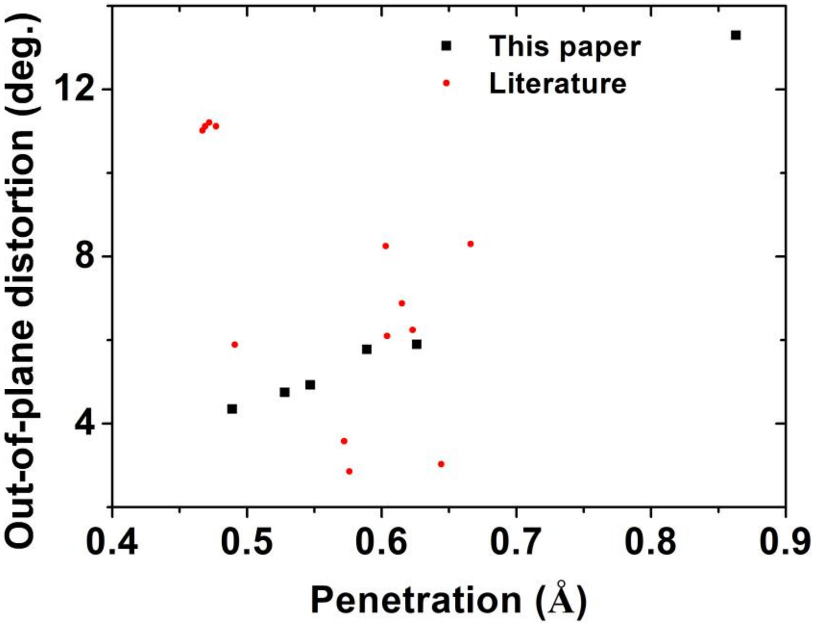

Fig. S6 Correlation between the out-of-plane distortion and nitrogen atom penetration depth from lead-iodide-based layered perovskites from this paper and previous reports.[4-7]

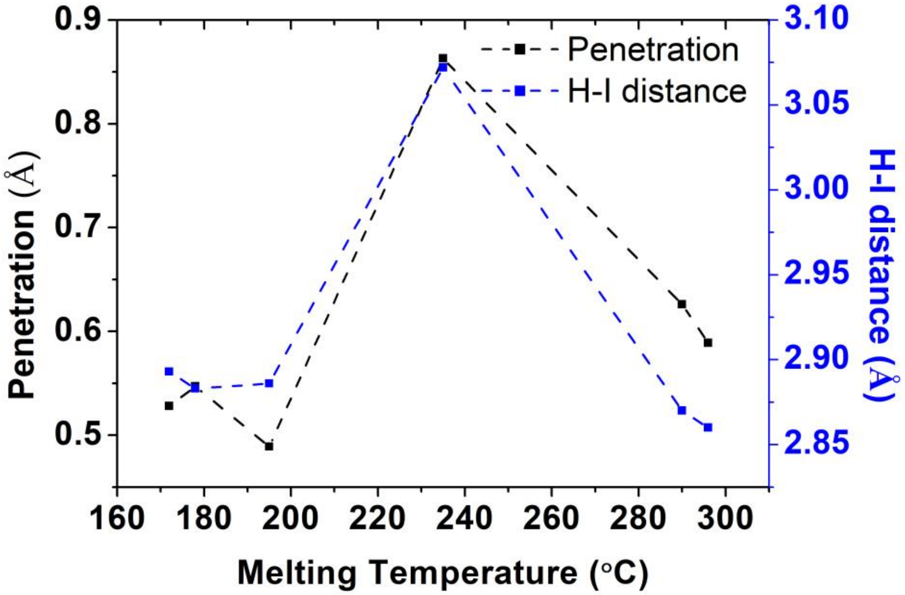

Fig. S7 Correlations between N penetration depth or average H-I distances of three closest H-I distances for hydrogen bonding and melting temperature of the six compounds discussed in this work.

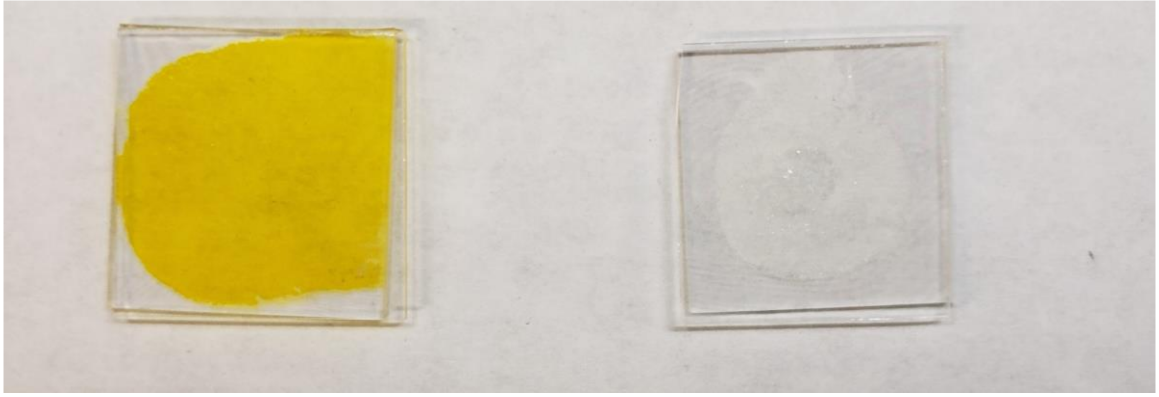

Fig. S8 Pictures of the melt processed films of (1-Me-ha)$_2$PbI$_4$ (left) and (1-Me-ha)$_2$PbBr$_4$ (right) between two glass substrates (1 inch X 1 inch) by melting and pressing the substrates on a hotplate.

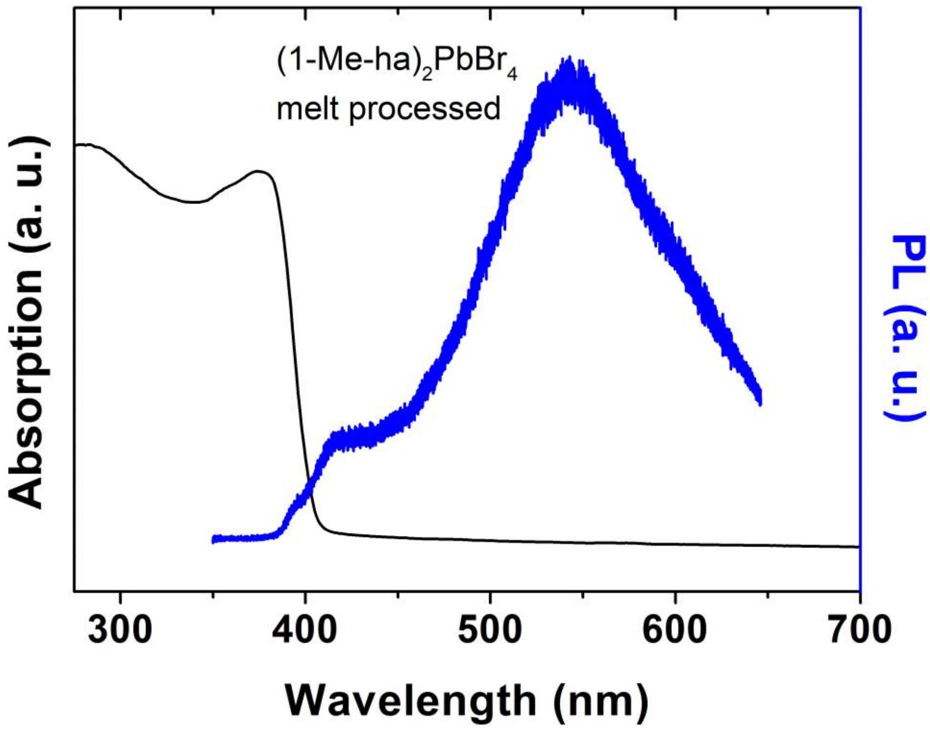

Fig. S9 UV-vis absorption and PL measurements of the melt-processed (1-Me-ha)$_2$PbBr$_4$ films.

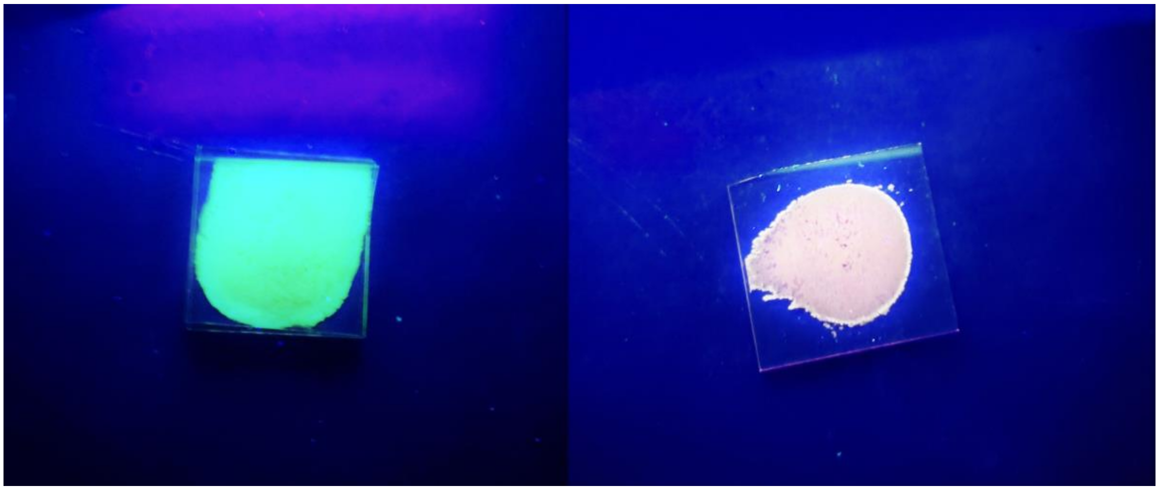

Fig. S10 Pictures of the melt processed films (as in Fig. S8) of $(1\text{-Me-ha})_2PbI_4$ (left) and $(1\text{-Me-ha})_2PbBr_4$ (right) under UV light.

Reference

1. O. V. Dolomanov, L. J. Bourhis, R. J. Gildea, J. A. K. Howard and H. Puschmann, *J. App. Cryst.*, 2009, **42**, 339-341.
2. G. Sheldrick, *Acta. Cryst. A*, 2015, **71**, 3-8.
3. S. Gonzalez-Carrero, G. M. Espallargas, R. E. Galian and J. Pérez-Prieto, *J. Mater. Chem. A*, 2015, **3**, 14039-14045.
4. K.-z. Du, Q. Tu, X. Zhang, Q. Han, J. Liu, S. Zauscher and D. B. Mitzi, *Inorg. Chem.*, 2017, **56**, 9291-9302.
5. D. G. Billing and A. Lemmerer, *New J. Chem.*, 2008, **32**, 1736-1746.
6. A. Lemmerer and D. G. Billing, *Dalton Trans.*, 2012, **41**, 1146-1157.
7. M. D. Smith, L. Pedesseau, M. Kepenekian, I. C. Smith, C. Katan, J. Even and H. I. Karunadasa, *Chem. Sci.*, 2017, **8**, 1960-1968.
